# Development and Mechanistic Insight into the Enhanced Cytotoxic Potential of Parvifloron D Albumin Nanoparticles in EGFR-Overexpressing Pancreatic Cancer Cells

**DOI:** 10.3390/cancers11111733

**Published:** 2019-11-05

**Authors:** Ana Santos-Rebelo, Pradeep Kumar, Viness Pillay, Yahya E. Choonara, Carla Eleutério, Mariana Figueira, Ana S. Viana, Lia Ascensão, Jesús Molpeceres, Patrícia Rijo, Isabel Correia, Joana Amaral, Susana Solá, Cecília M. P. Rodrigues, Maria Manuela Gaspar, Catarina Pinto Reis

**Affiliations:** 1 CBIOS (Research Center for Biosciences and Health Technologies), Universidade Lusófona de Humanidades e Tecnologias, Campo Grande 376, 1749-024 Lisboa, Portugal; ana.rebelo1490@gmail.com (A.S.-R.); p1609@ulusofona.pt (P.R.); 2 Department of Biomedical Sciences, Faculty of Pharmacy, University of Alcalá, Ctra. A2 km 33,600 Campus Universitario, 28871 Alcalá de Henares, Spain; jesus.molpeceres@uah.es; 3 Wits Advanced Drug Delivery Platform Research Unit, Department of Pharmacy and Pharmacology, Faculty of Health Sciences, School of Therapeutics Sciences, University of the Witwatersrand, Johannesburg, 7 York Road, Parktown 2193, South Africa; pradeep.kumar@wits.ac.za (P.K.); viness.pillay@wits.ac.za (V.P.); yahya.choonara@wits.ac.za (Y.E.C.); 4 Faculdade de Farmácia, Universidade de Lisboa, Av. Prof. Gama Pinto, 1649-003 Lisboa, Portugal; carlavania@ff.ul.pt (C.E.); mariana.selas.figueira@gmail.com (M.F.); 5 CQB, CQE, Faculdade de Ciências, Universidade de Lisboa, Campo Grande 1749-016 Lisboa, Portugal; apsemedo@fc.ul.pt; 6 CESAM, Universidade de Lisboa, Faculdade de Ciências, Campo Grande 1749-016 Lisboa, Portugal; lmpsousa@fc.ul.pt; 7 Research Institute for Medicines (iMed.ULisboa), Faculty of Pharmacy, Universidade de Lisboa, Av. Prof. Gama Pinto, 1649-003 Lisboa, Portugal; jamaral@ff.ulisboa.pt (J.A.); susana.sola@ff.ulisboa.pt (S.S.); cmprodrigues@ff.ulisboa.pt (C.M.P.R.); mgaspar@ff.ulisboa.pt (M.M.G.); 8 Centro de Química Estrutural, Instituto Superior Técnico, Departamento de Engenharia Química, Universidade de Lisboa,1049-001 Lisboa, Portugal; icorreia@tecnico.ulisboa.pt; 9 IBEB, Faculdade de Ciências, Universidade de Lisboa, 1749-016 Lisboa, Portugal

**Keywords:** pancreatic cancer, parvifloron D, nanoparticles, albumin, erlotinib

## Abstract

Pancreatic cancer is one of the most lethal cancers, with an extremely poor prognosis. The development of more effective therapies is thus imperative. Natural origin compounds isolated from *Plectranthus* genus, such as parvifloron D (PvD), have cytotoxic and antiproliferative activity against human tumour cells. However, PvD is a very low water-soluble compound, being nanotechnology a promising alternative strategy to solve this problem. Therefore, the aim of this study was to optimize a nanosystem for preferential delivery of PvD to pancreatic tumour cells. Albumin nanoparticles (BSA NPs) were produced through a desolvation method. Glucose cross-linking and bioactive functionalization profiles of BSA platform were elucidated and analysed using static lattice atomistic simulations in vacuum. Using the optimized methodology, PvD was encapsulated (yield higher than 80%) while NPs were characterized in terms of size (100–400 nm) and morphology. Importantly, to achieve a preferential targeting to pancreatic cancer cells, erlotinib and cetuximab were attached to the PvD-loaded nanoparticle surface, and their antiproliferative effects were evaluated in BxPC3 and Panc-1 cell lines. Erlotinib conjugated NPs presented the highest antiproliferative effect toward pancreatic tumour cells. Accordingly, cell cycle analysis of the BxPC3 cell line showed marked accumulation of tumour cells in G1-phase and cell cycle arrest promoted by NPs. As a result, erlotinib conjugated PvD-loaded BSA NPs must be considered a suitable and promising carrier to deliver PvD at the tumour site, improving the treatment of pancreatic cancer.

## 1. Introduction

Pancreatic cancer remains one of the most lethal cancers worldwide [1]. Conventional therapy approaches such as surgery, radiation and chemotherapy have had discrete impact, as approximately 100% of patients develop metastases and end up dying [1]. Chemotherapy is still the most common option, although with minimal impact on survival [2,3,4]. The current first-line chemotherapy agent in pancreatic cancer is gemcitabine, which extends the overall survival by only 6 to 12 weeks [3,5,6]. Additionally, its benefits are generally compromised by a low half-life and relative low concentration around the tumour tissue [7]. Therefore, there is an urgent need for novel and more effective therapies [1]. 

Nowadays, approximately 60% of clinically used antitumor drugs are derivatives or come directly from natural products [8,9]. Terpenoids are the largest class of natural products with medicinal properties and they represent a rich source for drug discovery, especially anticancer drugs [10]. The majority induce tumour cell death by targeting apoptotic pathways [10]. Abietane diterpenoids are characteristic secondary metabolites from the Lamiaceae family reported to have cytotoxic and antiproliferative activity against tumour cell lines [11]. More specifically, *Plectranthus ecklonii* and its major compound, parvifloron D (PvD), caught our attention [12,13]. PvD has been shown to inhibit cancer proliferation by apoptosis in some cancer cell lines, such as human myeloid leukaemia, melanoma and breast cancer [12]. However, PvD presents very poor water solubility characteristics, as well as an apparent lack of selectivity toward cancer cells [12,14]. Since PvD also shows lack of specificity and cytotoxicity in noncancerous cell lines, the use of nanotechnology appears as a possible solution to deliver this drug to pancreatic cancer tissue without undesirable side effects. 

Besides improving solubility and stability, nanoparticles (NPs) may extend formulation actions, combine activities with different degrees of hydrophilicity/lipophilicity, crucial for targeting and to deliver the drug at a specific tissue or organ [15,16,17]. In addition, NPs allow a better action of natural products, promoting a sustained release with reduced dose administration [15,18,19]. In this regard, albumin NPs (BSA NPs) are increasingly being used as drug delivery system for effective accumulation within tumour tissues through the enhanced permeability and retention (EPR) effect and albumin binding target proteins [6,20]. In fact, albumin is the most abundant protein in blood plasma, being biocompatible, biodegradable and nontoxic [6,20,21,22], and exhibiting active targeting and specific activity in the liver-pancreas system [7,23]. 

It has been shown that specific targeting of tumour cells can be guaranteed through different linkers or functional molecules [24,25]. Polyethylene glycol (PEG) is one of the most used polymers [26,27] since PEGylation has been shown to reduce NP immunogenicity and enhance accumulation in tumours by heightening the circulation time and promoting the EPR effect [24,27]. Further, the addition of antibodies, like cetuximab (CET) and erlotinib (ERL), was already shown to be necessary to promote targeted delivery. Indeed, the Epidermal Growth Factor Receptor (EGFR) is overexpressed in pancreatic cancer and both antibodies are EGFR inhibitors [28].

Therefore, due to the low water-solubility of PvD, we have encapsulated PvD into a biocompatible and hydrophilic nanomaterial as a possible drug delivery system. Bovine serum albumin (BSA) was chosen as encapsulating material. In order to produce our BSA NPs, a desolvation method was used. This method was chosen given its advantages, such as the fact that it does not require a temperature increase, being a suitable method for heat sensitive polymers, like BSA [29,30]. In the last step of desolvation method, glutaraldehyde is the most common added cross-linking agent [21,31,32]. However, due to glutaraldehyde’s undesirable effects, we have tested more biocompatible alternative cross-linking methods including ultraviolet (UV) irradiation, addition of glucose, and combinations of both. Finally, to further optimize the NP preparation method, other different conditions were tested, including different stirring rates during the emulsion step (100 and 500 rpm), cross-linking times (30 min and 24 h) and type of organic solvent (hexane, acetone, DMSO and ethanol), and aqueous:organic solvent proportions (1:1, 2:1 and 3:1). The organic solvents tested were chosen for being the ones that best dissolve our compound with different ICH classification, i.e., grade of toxicity. All organic solvents were properly removed with centrifuge wash cycles after NP production. 

To summarize, in the present work we produced PvD-loaded BSA NPs followed by their functionalization with ERL and/or CET for pancreatic cancer cell targeting.

## 2. Results

### 2.1. Optimization of BSA-Based NPs Preparation Method

To optimize the NP preparation method, different conditions were tested to select the best conditions to achieve BSA NPs with a mean size ranging between 100–400 nm, monodisperse size distribution, high cross-linking efficacy and the lowest negative zeta potential, to ensure high stability [33]. Table 1 shows the results obtained with all the conditions tested.

After analysing the designed particles in terms of mean size, zeta potential and cross-linking efficacy [CE (%)], and testing different rpm (100 and 500 rpm), we conclude that the best outcomes were achieved with a stirring rate of 500 rpm with smaller particle size and lower PdI. Then, for the four cross-linking tested groups, the cross-linking with glucose showed the smallest size. However, when observing the cross-linking time (30 min and 24 h), we choose 30 min as the best cross-linking time, since the CE% drastically decreased over the time. Hereupon, comparing the results obtained for all organic solvents tested, we have elected acetone. Indeed, acetone is a ICH Class 3, i.e., regarded as less toxic and of lower risk to human health, and, of note, class 3 includes no solvent known as a human health hazard at levels normally accepted in pharmaceuticals. Finally, we tested different proportions of aqueous BSA: organic solvent ratios, and choose the 3:1. The resulting particle size was near 200 nm, presenting a negative zeta potential and high CE (up to 90%), close to the established goal. In bold (Table 1), is represented the best result in each condition tested.

### 2.2. Cytotoxicity Assay in a Saccharomyces Cerevisiae Model 

**Figure 1:**
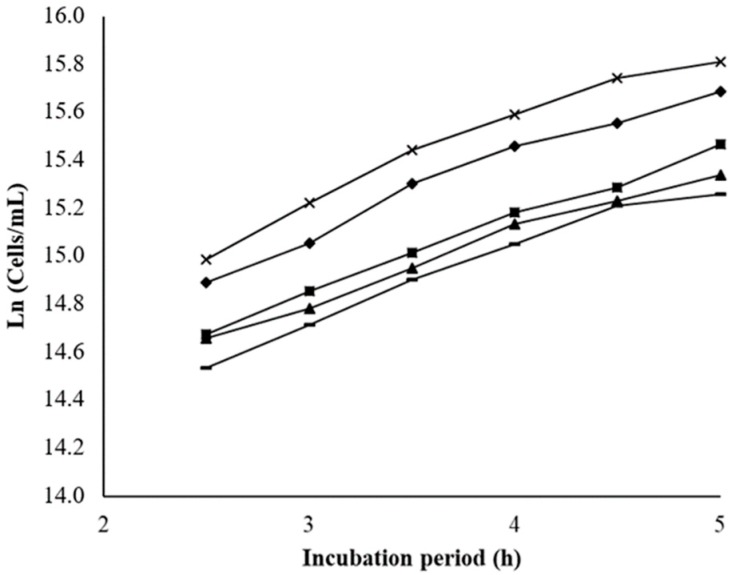
Growth curves of *Saccharomyces cerevisiae* cultures exposed to BSA NPs (**x** - negative control; ♦ - 3.0 μM; ■ - 3.9 μM; ▬ - 4.6 μM; ▲ - 5.3 μM). Yeast growth is presented as the natural logarithm of cell concentration, which is expressed as number of yeast cells/mL. The interval 2–5 h was considered as the log growth phase (n = 4, mean ± SD).

The percentage of growth inhibition (GI) was calculated by the determination of the linear slope in the logarithmic phase for each sample (Figure 1). 

Interestingly, the exposure to BSA NPs at the lowest concentration (3.0 μM) resulted in 3.2% of GI and at the highest concentration (5.3 μM) resulted in 15.6%. On the other hand, the negative control was assumed as producing zero percentage of GI (100% yeast growth), in each experiment.

### 2.3. Circular Dichroism Spectroscopy

Circular dichroism spectroscopy was used to analyse the secondary structure of the BSA protein in its native form (BSA), NPs (BSA NPs) and NPs with ERL (ERL conjugated BSA NPs). Samples of NPs that were randomly destroyed by ultra-sounds and heat (Destroyed NP) were also analysed to confirm if this method destroys the BSA secondary structure and consequently NPs. 

**Figure 2:**
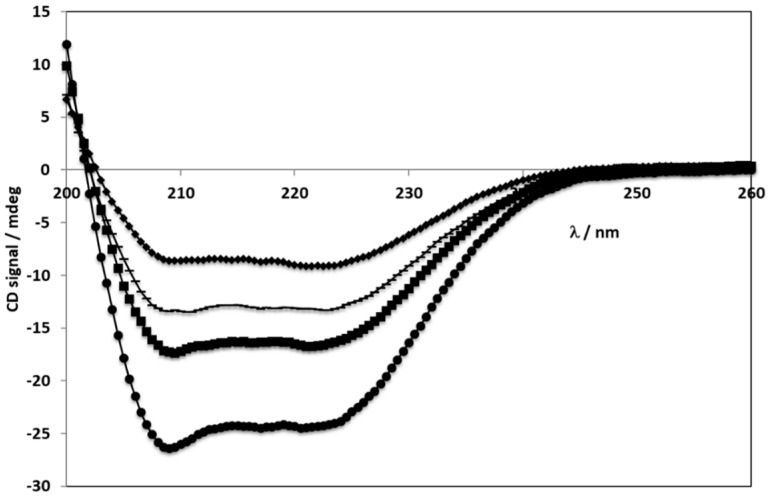
CD spectra measured in the far UV range for all samples: ● - BSA 0.2 mg/mL; ♦ - BSA NPs 0.4 mg/mL; *■* - ERL conjugated BSA NPs 0.4 mg/mL; *▬ -* Destroyed NPs 0.4 mg/mL.

**Figure 3:**
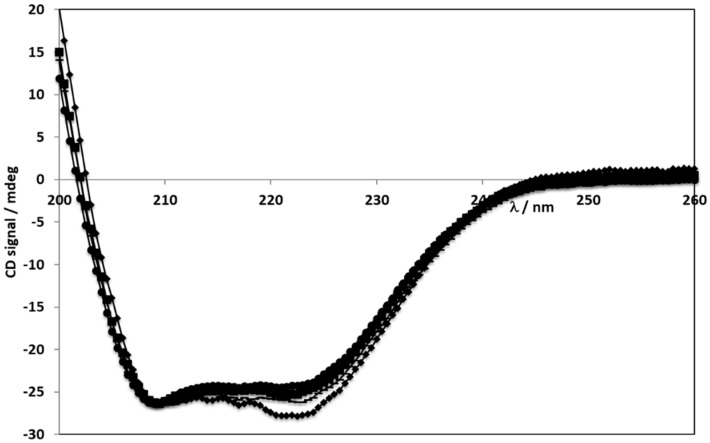
CD spectra measured in the far UV range after normalization – all spectra having the same intensity as BSA (● - BSA 0.2 mg/mL; ♦ - BSA NPs 0.4 mg/mL; *■* - ERL conjugated BSA NPs 0.4 mg/mL; ▬ - Destroyed NPs 0.4 mg/mL).

All samples were quantified in water and the respective spectra were measured in the far UV with protein concentrations corresponding to 0.2 or 0.4 mg/mL. Figure 2 and Figure 3 show the collected spectra. Typically, α-helix showed two maxima of negative signal at 208 and 222 nm. Antiparallel β-sheet shows one negative maximum at ca. 218 nm and a positive one at ca. 195 nm. Random coil is characterized by a negative maximum at ca. 208 nm.

Circular dichroism has been extensively applied to evaluate the secondary structure of proteins as different amounts of secondary structural elements, such as α-helix and β-sheets, correspond to different CD spectra. This is due to the protein CD spectrum being considered the sum of the spectra of the individual secondary structures present in the protein. In order to have the same intensity as the BSA spectrum, the spectra of all complexes in Figure 3 were multiplied by a constant.

BSA showed a typical CD spectrum with a high α-helical content - negative ellipticity at ~208 and ~220 nm in the far UV range. After aggregation and formation of the NPs the spectra lost intensity but kept their shape. This might indicate one of two possible effects, either the α-helical content decreased, or the protein concentration is lower. Without having a control experiment to accurately determine the protein concentration no conclusions can be made. However, the persistence of the band at 220 nm suggests that it is only a concentration decrease. Thus, the preparation process used to destroy the NP (ultra-sounds and increased heat) did not destroy the secondary structure of BSA, revealing that those methods are safe and do not affect BSA structure. Importantly, BSA secondary structure is maintained even when in particle form [34,35].

### 2.4. Hemolytic Activity of Potential Injectable PvD-loaded BSA NPs Dosage Form

Understanding the interactions between NPs and blood components is critical for future clinical applications. One of the most fundamental studies on the interactions between NPs and blood components is to access their haemolytic properties. In fact, in vivo haemolysis can trigger several pathological conditions such as anaemia and jaundice. in vitro evaluation of NPs biocompatibility is a crucial step towards early preclinical development because the small size and unique physicochemical properties of NPs may cause damage in red blood cells (RBC). Thus, the US FDA recommends that an in vitro haemolysis study should be performed for compounds intended for injectable use, at the intended concentration for i.v. administration to test the possible haemolytic potential. This assay evaluates haemoglobin release in the plasma, as an indicator of RBC lysis, following the exposure to a test agent [36]. In this work, the haemolytic activity against RBCs was used as a marker of a general membrane toxicity effect of PvD. Since the concentration of haemoglobin depends both on the number of disintegrated RBCs in the sample and the volume of the fluid, the degree of haemolysis is often described as the percentage of free haemoglobin in relation to the total. The percentage of haemolysis of PvD in free form or incorporated in BSA NPs, as well as in empty NPs, was evaluated for concentrations up to 50 μM. For all the formulations at the tested concentrations, the haemolysis was always below 1%, ensuring their safety for i.v. administration. 

### 2.5. PvD Encapsulation Efficiency 

The encapsulation efficiency (EE, %) is one of the most important physicochemical characteristics of NPs-based formulations. Many methods have been used to evaluate the EE, including dialysis bag diffusion, gel filtration, ultrafiltration, and ultracentrifugation. Compared with the conventional drug delivery systems, the size of the reservoir of NPs in which the drug molecule is loaded is extremely small. Depending on this small capacity of NPs, the drug molecules interact with the polymeric structure to a certain degree. In this work, EE (%) was determined for all nanoformulations by measuring the non-encapsulated drug (lost PvD during the nanoencapsulation procedure) by centrifugation, i.e., through indirect quantification. Herein, we obtained 79.47%, 82.07%, 79.92%, 72.76%, for PvD-loaded BSA NPs, ERL conjugated PvD-loaded BSA NPs, CET conjugated PvD-loaded BSA NPs and ERL-CET conjugated PvD-loaded BSA NPs, respectively. For this assay, a calibration curve was firstly performed using previously isolated PvD as a standard. PvD solutions ranging from 4.4 to 44 μg/mL were evaluated and a calibration curve (y = 31316x − 66339) was obtained with R^2^ = 0.999. Limit of Detection (LOD) and Limit of Quantification (LOQ) were calculated to be 2.2 μg/mL and 6.5 μg/mL, respectively.

### 2.6. ERL and CET Conjugated BSA NPs Conjugation Quantification

The ideal NP-based formulations should have a specific targeting to tumour cells, minimizing or even avoiding off-target effects of the drug on healthy tissues. In this field, much research has conjugated targeting ligands specific to cell surface components that are unique or upregulated in tumour tissues to NPs surfaces. In the current study, the presence of carboxylic and amino groups on the surface promotes the surface functionalization for BSA NPs. The ERL and CET conjugation quantification was determined by measuring the non-conjugated ligand, i.e., again through indirect quantification. The value was 40 µg/mL, for ERL conjugated PvD-loaded BSA NPs, ERL conjugated empty BSA NPs and ERL-CET conjugated empty BSA NPs, and 0.01 µg/mL for ERL-CET conjugated PvD-loaded BSA NPs. CET conjugated values were equivalent to those measured for ERL conjugation.

### 2.7. Static Lattice Atomistic Simulations (SLAS) in Vacuum

#### 2.7.1. Molecular Mechanics Assisted Model Building and Energy Refinements

Molecular mechanics energy relationship (MMER), a method for analytical-mathematical representation of potential energy surfaces, was used to provide information about the contributions of valence terms, noncovalent Coulombic terms, and noncovalent van der Waals interactions for the BSA/cross-linker and BSA/bioactive interactions. The MMER model for potential energy factor in various molecular complexes can be written as follows in Equation (1):

**E_molecule/complex_** = V_∑_ = V_b_ + V_θ_ + V_φ_ + V_ij_ + V_hb_ + V_el_ (1)

where, V_∑_ is related to total steric energy for an optimized structure, V_b_ corresponds to bond stretching contributions, V_θ_ denotes bond angle contributions, V_φ_ represents torsional contribution from dihedral angles, V_ij_ incorporates van der Waals interactions due to non-bonded interatomic distances, V_hb_ symbolizes hydrogen-bond energy function and V_el_ stands for electrostatic energy.

In addition, the total potential energy deviation, ΔE_Total_, was calculated as the difference between the total potential energy of the complex system and the sum of the potential energies of isolated individual molecules, as in the following Equation (2):

**ΔE_Total(A/B)_** = E_Total(A/B)_ - (E_Total(A)_ + E_Total(B)_) (2)

The molecular stability can then be estimated by comparing the total potential energies of the isolated and complexed systems. If the total potential energy of complex is smaller than the sum of the potential energies of isolated individual molecules in the same conformation, the complexed form is more stable and its formation is favoured [37].

#### 2.7.2. Fabrication of Glucose Cross-Linked BSA NPs

**Figure 4:**
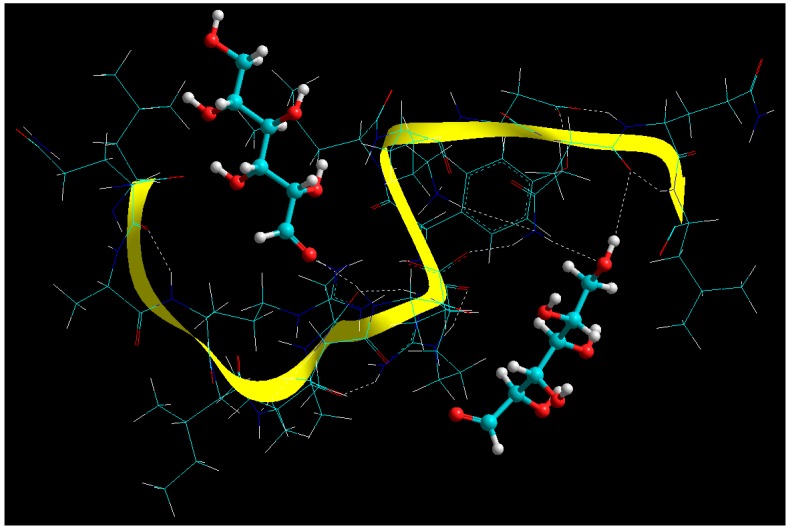
Visualization of geometrical preferences and functional interactions between BSA (stick rendering) in complexation with the glucose molecules (ball-and-tube rendering) after molecular mechanics simulations in vacuum. Colour codes: C (cyan), O (red), N (blue) and H (white).

The energy relationships confirming the cross-linking of BSA with glucose are presented in Equations (3)–(5) and the corresponding energy minimized geometrical conformations are depicted in Figure 4:

**E_BSA_** = −276.821*V_∑_* = 4.001*V_b_* + 20.569*V_θ_* + 29.478*V_φ_* − 23.961*V_ij_* − 6.478*V_hb_* − 300.432*V_el_* (3)

**E_GLUCOSE_** = 1.064*V_∑_* = 0.556*V_b_* + 2.998*V_θ_* + 1.696*V_φ_* + 6.820*V_ij_* − 0.000*V_hb_* + 11.008*V_el_* (4)

**E_BSA-GLUCOSE_** = −342.611*V_∑_* = 5.383*V_b_* + 26.787*V_θ_* + 38.058*V_φ_* − 21.609*V_ij_* − 4.323*V_hb_* − 386.908*V_el_* (5)

**Figure 5:**
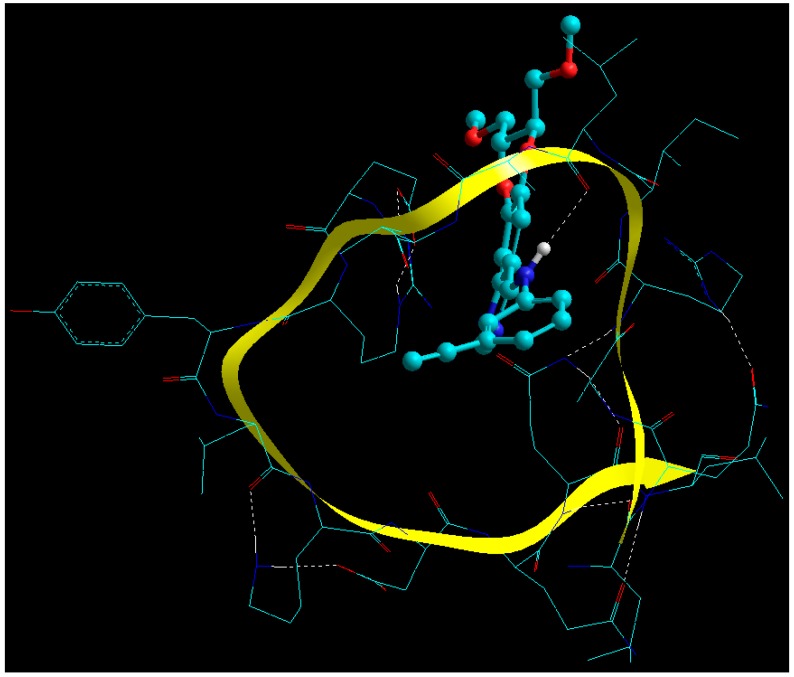
Visualization of geometrical preferences and functional interactions between BSA (stick rendering) in complexation with the ERL molecule (ball-and-tube rendering) after molecular mechanics simulations in vacuum. Colour codes: C (cyan), O (red), N (blue) and H (white).

It is evident from MMER that BSA-glucose formed a very stable complex, with ΔE_BINDING_ of −66.854 kcal/mol, wherein a major stabilizing role was played by electrostatic interactions (ΔE = −97.484 kcal/mol) and partially by van der Waals forces (ΔE=−4.468 kcal/mol). As expected from a cross-linked structure, the torsional strains caused by a small molecule cross-linking agent such as glucose may cause an internal stress arising from bond/angle stretching and bending, leading to a rigid structure and hence destabilization of the matrix as evident from the bonding energy terms viz *V_b_* (ΔE = 0.826 kcal/mol)*, V_θ_* (ΔE = 3.22 kcal/mol) and *V_φ_* (ΔE = 6.884 kcal/mol). Interestingly, such constrained structure will bring the subpeptidic chains closer and hence enhancing the electrostatic interactions contributing to the final stabilized structure. It is worth noting that the H-bonding component of BSA was somewhat diminished (ΔE = 2.155 kcal/mol) after cross-linking, which may be due to reduction in intramolecular H-bonding and introduction of intermolecular H-bonding. This observation is further confirmed by Figure 5 wherein: (1) the reducing sugar-derived carbonyl group of glucose formed an adduct with free amine group of BSA (–C=O…NH–) [38]; (2) –OH…O=C– hydrogen bonding; and (3) -OH…NH– hydrogen bonding [39]. These findings further corroborate with the experimental data wherein it was proposed that longer cross-linking time may lead to reduced cross-linking efficiency which may further be attributed to the above inter- and intra-molecular H-bonding interplay.

#### 2.7.3. Functionalization of BSA NPs with ERL 

The geometrical visualization and MMER profiles for BSA-ERL complex are depicted in Equations (6)–(8) and Figure 5, respectively:

**E_BSA_** = −276.821*V_∑_* = 4.001*V_b_* + 20.569*V_θ_* + 29.478*V_φ_* − 23.961*V_ij_* − 6.478*V_hb_* − 300.432*V_el_* (6)

**E_ERL_** = 17.719*V_∑_* = 0.995*V_b_* + 1.991*V_θ_* + 5.833*V_φ_* + 8.899*V_i_* (7)

**E_BSA-ERL_** = −284.457*V_∑_* = 5.650*V_b_* + 45.119*V_θ_* + 53.256*V_φ_* − 37.967*V_ij_* − 7.469*V_hb_* − 343.046*V_el_* (8)

The aim of generating MMER in this case was to confirm the mode of interaction inherent to the peptide-bioactive complex. In case of bioactives’ functionalization in a carrier, it is of utmost important that the functional groups responsible for their bioactivity are not involved in covalent bonding. In the current modelling exercise, it was established that the N atoms of ERL were H-bonded to the -C=O functionality of BSA. This is in line with previously developed ERL-functionalized carriers by Ali and co-workers, one of the first reports mentioning such functionalization [40]. The BSA-ERL complex was energetically stabilized with ∆E_binding_ of −25.355 kcal/mol with both bonding and non-bonding interactions playing a significant part. The significant destabilization by the bonding interactions (*V_b_, V_θ_* and *V_φ_*) led to significant geometrical changes in the BSA molecule further leading to apparent globalization which may further assist in *colloidation* of the nanosystem. The 41.158 kcal/mol destabilization by the bonding interactions was neutralized and exceeded by the non-bonding stabilization of 66.51 kcal/mol with electrostatic interactions playing the major role. Interestingly the close fit of the ERL molecule further stabilized the van der Waals contributions and hence the H-bonding.

#### 2.7.4. Functionalization of BSA NPs with CET 

In a study published in *PNAS*, Donaldson and co-workers provided an interesting proposition that can be quoted as follows “the identification and subsequent grafting of a unique peptide binding site within the Fab domain offers a unique means of adding functionality to monoclonal antibodies through a noncovalent interaction including improved pre-targeted imaging, alternative payload delivery, and cross-linking of mAbs on cell surfaces to enhance their therapeutic potential” [40]. Taking lead from the above proposition, the current study employed CET as the mAb and tested the potential of grafting BSA to the light (A, C) and heavy (B, D) chains of CET. As this study doesn’t involve molecular docking, the light and heavy chains were individually interacted with the BSA molecule and thereby light chain A was selected for the final modelling paradigm based on preliminary geometrical and energetic interaction profiling (detailed docking studies are out-of-scope of this study). Similar to ERL, the BSA-CET molecular complex was destabilized by the bonding energy terms (bond, angle, and torsion) while significantly stabilized by the non-bonding energy components (van der Waals forces, H-bonding, and electrostatic interactions) as depicted in Equations (9)–(11):

**E_BSA_** = −276.821*V_∑_* = 4.001*V_b_* + 20.569*V_θ_* + 29.478*V_φ_* − 23.961*V_ij_* − 6.478*V_hb_* − 300.432*V_el_* (9)

**E_CET_** = −959.541*V_∑_* = 17.270*V_b_* + 124.263*V_θ_* + 123.955*V_φ_* − 167.367*V_ij_* − 34.565*_hb_* − 1023.1*V_el_* (10)

**E_BSA-CET_** = −1281.649*V_∑_* = 23.443*V_b_* + 237.799*V_θ_* + 203.402*V_φ_* − 238.943*V_ij_* − 44.723*V_hb_* − 1462.63*V_el_* (11)

**Figure 6:**
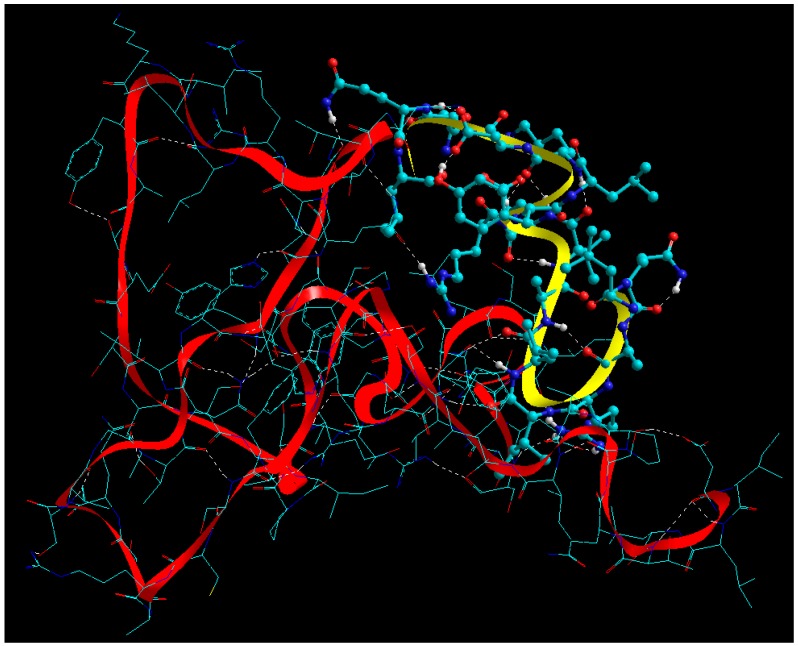
Visualization of geometrical preferences and functional interactions between BSA (yellow ribbon rendering) in complexation with the CET Chain A (red ribbon rendering) after molecular mechanics simulations in vacuum. Colour codes: C (cyan), O (red), N (blue) and H (white).

A close look at Figure 6 revealed an extensive molecular network with –C=O…N-H amide linkages and –C=O…O-H hydrogen bonds.

### 2.8. Physical and Morphological Characterization of the EGFR Inhibitors Conjugated BSA NPs

Table 2 shows DLS analysis of particle size and zeta potential of ERL conjugated empty BSA NPs, ERL conjugated PvD-loaded BSA NPs, CET conjugated empty BSA NPs, CET conjugated PvD-loaded BSA NPs, ERL-CET conjugated empty BSA NPs, ERL-CET conjugated PvD-loaded BSA NPs, PvD-loaded BSA NPs and Empty BSA NPs.

**Figure 7:**
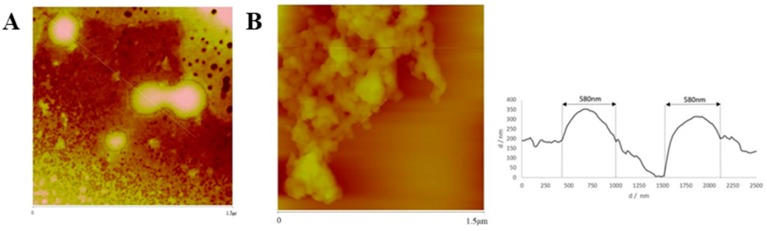
AFM images (2D and sectorial images) with size scale of (**A**) PvD-loaded, and (**B**) Empty ERL-CET conjugated NPs.

**Figure 8:**
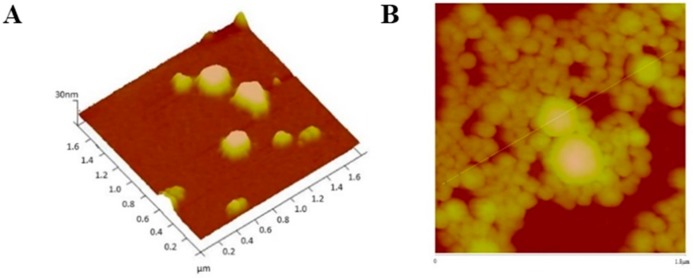
AFM images (3D and 2D images) with size scale of (**A**) PvD-loaded, and (**B**) empty ERL conjugated NPs.

**Figure 9:**
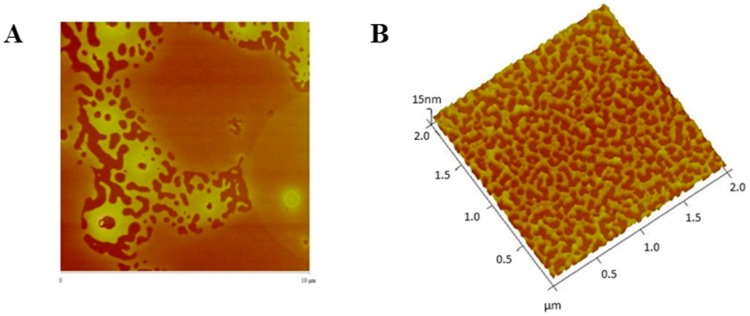
AFM images (2D and 3D images) with size scale of (**A**) PvD-loaded, and (**B**) empty CET conjugated NPs.

**Figure 10:**
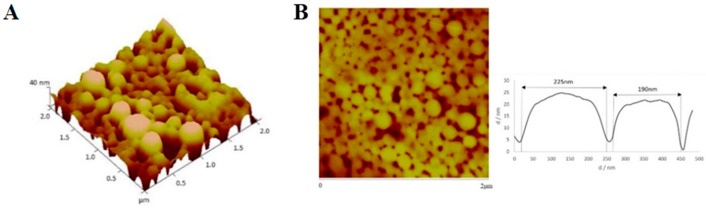
AFM images (3D and sectorial images) with size scale of (**A**) PvD-loaded, and (**B**) empty non-conjugated NPs.

AFM analysis confirmed that particles sizes were very close to those obtained by DLS analysis. Figure 7, Figure 8, Figure 9 and Figure 10 show AFM images of all formulations prepared as well as their size and morphology. 

**Figure 11:**
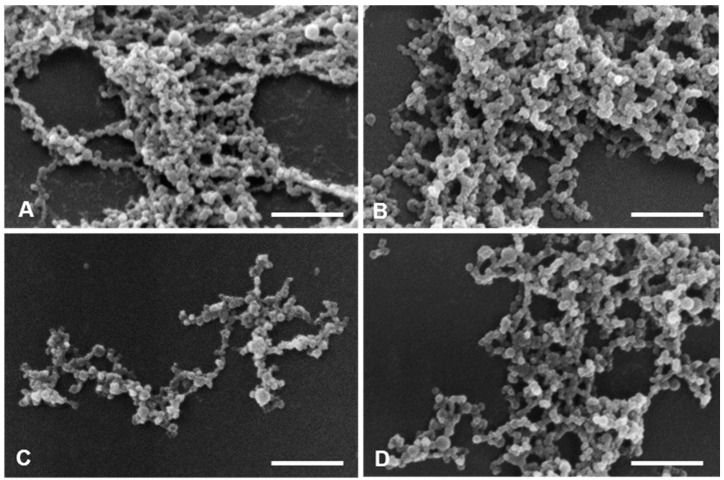
SEM images of BSA NPs. (**A**) empty ERL conjugated NPs; (**B**) PvD-loaded non-conjugated NPs; (**C**) ERL conjugated PvD-loaded NPs; (**D**) empty non-conjugated NPs. Scale bars = 1 μm.

**Figure 12:**
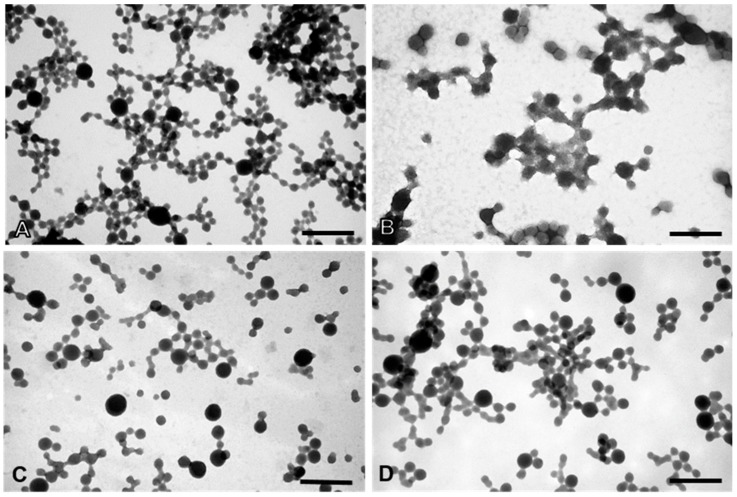
TEM images of BSA NPs. (**A**) empty ERL conjugated NPs; (**B**) PvD-loaded non-conjugated NPs; (**C**) ERL conjugated NPs PvD-loaded; (**D**) empty non-conjugated NPs. Scale bars = 0.5 μm.

AFM allows the optimization of biomaterials processes, performance, physical and chemical properties, offering a great contribution to the understanding of surface and interface properties, even at the nanoscale [41]. Considering DLS and AFM analysis, as well as the cell viability studies of PvD-loaded BSA NPs, we have decided to proceed only with the best particles in terms of mean size (between 100–400 nm, allowing a safe i.v. administration), zeta potential (higher than −30 mV, allowing a higher physical stability [33]), with monodisperse size distribution and lowest IC_50_ values as followed described. Thus, SEM and TEM focused on ERL conjugated NPs and non-conjugated NPs, confirming particle sizes and spherical morphology (Figure 11 and Figure 12).

### 2.9. Cell Viability Studies of PvD-loaded BSA NPs

Free PvD was shown highly cytotoxic for both cell lines, being particularly cytotoxic in BxPC3 cell line (Table 3). 

However, the IC_50_ of PvD-loaded BSA NPs was higher than 30 µM, suggesting that at the end of the 48 h of incubation, PvD was not totally released from the particles. In fact, we know from previous in vitro studies (Santos-Rebelo et al. [13]) that total PvD particle release only occurs after 72 h of incubation. Indeed, NPs are possibly inducing its expected effect, since it would not be desirable that PvD cytotoxicity occurs during the administration, but only in the target tumor site. Nevertheless, IC_50_ of ERL conjugated PvD-loaded BSA NPs and ERL-CET conjugated PvD-loaded BSA NPs are lower than those of non-conjugated particles. However, when comparing ERL conjugated empty BSA NPs and ERL-CET conjugated empty BSA NPs, the IC_50_ values are also low, which leads us to hypothesize that the toxicity observed for ERL conjugated PvD-loaded BSA NPs and ERL-CET conjugated PvD-loaded BSA NPs may results from the ERL on the NP surface. In fact, this can increase particle internalization, allowing a higher drug release inside the target cells, particularly in the BxPC3 cell line. In vivo assays are needed to confirm targeting in a living organism. 

### 2.10. Cell Cycle Assays

**Figure 13:**
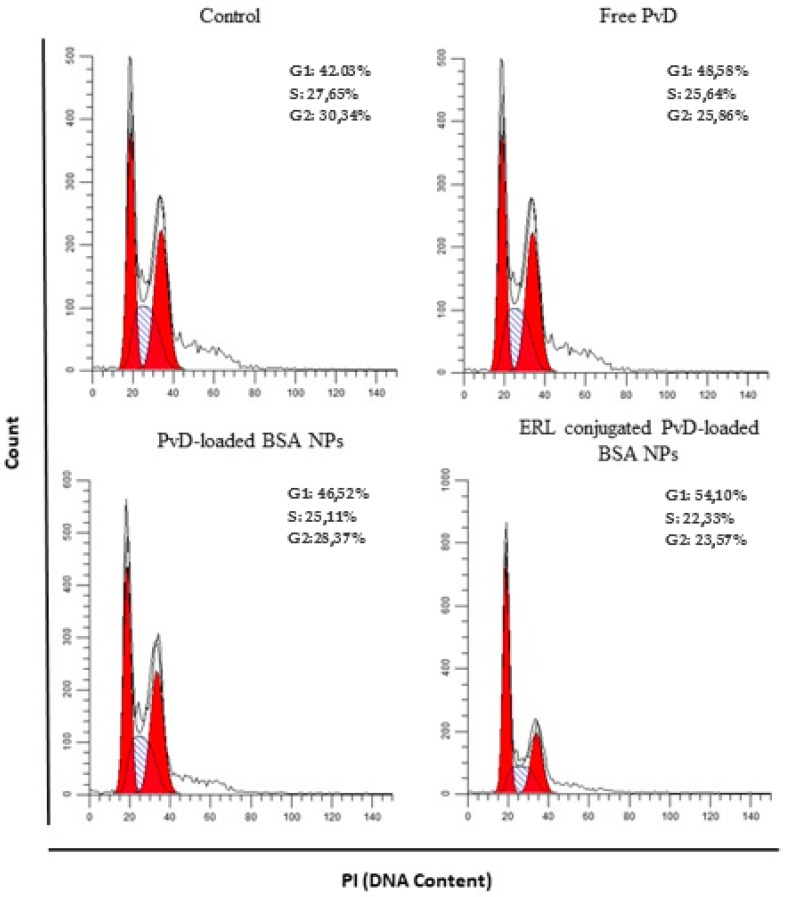
Effect of NPs on tumour cell cycle progression. Cellular DNA was stained with propidium iodide (PI) to determine cell cycle distribution. BxPC3 cells were treated with free PvD, PvD-loaded BSA NPs and ERL conjugated PvD-loaded BSA NPs, and compared with control, 24 h after incubation.

**Figure 14:**
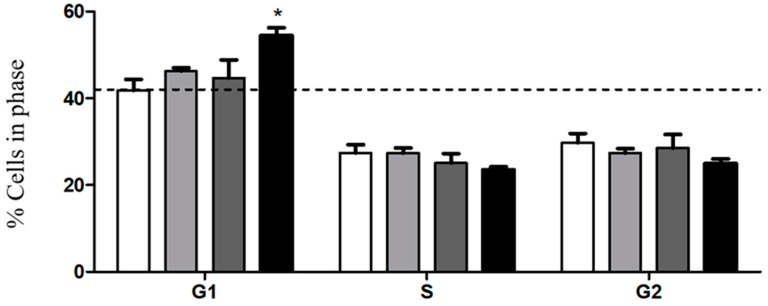
Effect of NPs on tumour cell cycle progression. Cellular DNA was stained with propidium iodide (PI) to determine cell cycle distribution. Percentage of cells in G1, S and G2 cell cycle phases in no addition (control; white), Free PvD (light grey), PvD-loaded BSA NPs (dark grey) and ERL conjugated PvD-loaded BSA NPs (black), after 24 h incubation. Results are expressed as mean ± SEM for three different experiments. * *p* < 0.05 from control by one-way ANOVA test.

To go deeper into the mechanisms by which these different particles impact on tumour cell proliferation, we evaluated cell cycle progression in BxPC3 cells after incubation with free PvD, PvD-loaded BSA NPs and ERL conjugated PvD-loaded BSA NPs, and comparing with a blank control in which cells grew without any chemical influence. Figure 13 and Figure 14 show the percentage of cells in different phases of the cell cycle after 24 h of particle incubation. As observed in Figure 14, exposure of cells to ERL conjugated PvD-loaded BSA NPs induced a marked accumulation of cells in the G1 phase, when compared with control (*p* < 0.05). Regarding PvD-loaded BSA NPs, the results show that these forms did not affect cell cycle progression. Importantly, this is in agreement with our previous hypothesis that total PvD is not totally released from the NPs at 24 h of incubation. Further, ERL in NPs surface probably increases particle internalization, allowing a higher drug release inside the target cells. In conclusion, these outcomes indicate that both PvD and ERL are responsible for driving tumour cells to arrest in the G1 phase of the cell cycle, preventing them from dividing and spreading.

### 2.11. Lactate Dehydrogenase Assay

**Figure 15:**
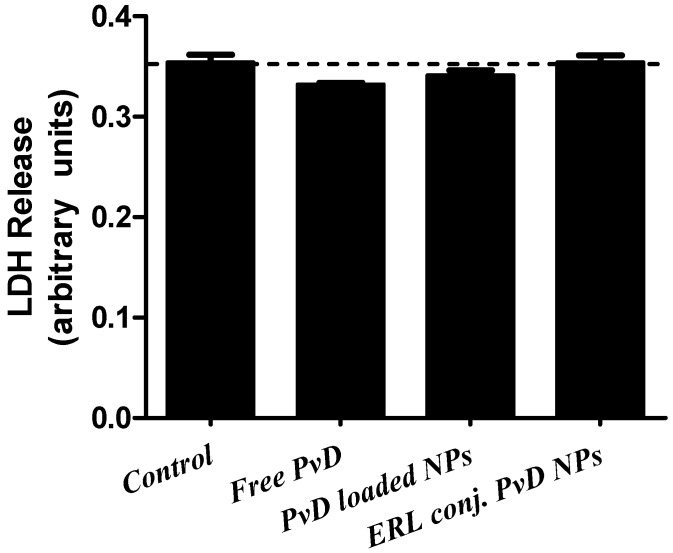
Lactate dehydrogenase (LDH) assay, using free PvD, ERL conjugated empty BSA NPs, ERL conjugated PvD-loaded BSA NPs. Results are expressed as mean ± SEM for three different experiments.

To evaluate if PvD-dependent decrease of cell viability was due to either cell death or cell cycle arrest, we assessed lactate dehydrogenase release as a measure of cell membrane disruption, and therefore cell death (Figure 15).

No significant differences were detected in membrane integrity between all samples tested, suggesting that the decrease of cell viability likely results from cell cycle arrest.

## 3. Discussion

Pancreatic cancer median survival after diagnosis ranges between 2–8 months, making this one of the most lethal types of cancer [2]. Moreover, the application of conventional drugs approved against pancreatic cancer is limited by drug resistance and a large number of adverse side effects [13]. Taking this into account, new therapies as nanoparticle applications could become a promising tool for sustained delivery of anticancer drugs.

We previously confirmed that PvD is a potential lead molecule mainly due to its specificity to pancreatic tumour cells [2]. However, PvD has very low water solubility and it strongly requires a suitable carrier like BSA NPs. In the present study, BSA NPs for PvD delivery were produced with an optimized method, where the conditions chosen include 500 rpm stirring rate, 30 min of cross-linking time and 3:1 aqueous/acetone ratio. These conditions resulted in particles with particle size ranging between 100–400 nm, monodisperse size distribution, high CE and negative zeta potential. BSA NPs were tested for toxicity in a *Saccharomyces cerevisiae* model, showing no relevant toxicity in the highest concentration tested, indicating promising carrier characteristics to deliver our drug. CD spectroscopy revealed the main structure of our NPs, where BSA secondary structure remained intact after nanoparticle arrangement. Previous studies by Differential Scanning Calorimetry [13] suggest that following NPs arrangement the structure modification of BSA chains may not involve changes in the protein secondary structure. Further, BSA secondary structure, even after NPs arrangement, seems to resist to heat and ultra-sounds. Finally, haemolytic activity was tested in BSA NPs as well as in free drug, to ensure that our formulation could be applied by i.v. administration. Since the haemolytic activity was below 1%, even for the highest concentrations tested, our formulation may be considered safe for i.v. administration, without any haemolytic risk.

The promising formulation, here developed, presented an EE greater than 80%, a value that is very high in comparison with other drug delivery systems. For example, in some micellar NPs the maximum capacity of the drug is only around 20–30% [42].

To target NPs to pancreatic cancer cells, we used EGFR inhibitors, ERL and CET, since EGFR overexpression has been found in 90% of pancreatic cancers [2]. NP conformation was further elucidated using SLAS in vacuum. Most studies with BSA NPs have used glutaraldehyde as cross-linking agent [43]. However, glutaraldehyde demonstrated toxicity to healthy cells [43]. We have proposed to use glucose as cross-linking agent and MMER confirmed that BSA-glucose formed a very stable complex. Moreover, MMER profiles for BSA-ERL complex showed that the functional groups responsible for bioactivity are not involved in covalent bonding, leaving them free to ensure their action in targeting tumour cells, and yet the BSA-ERL complex was considered energetically stabilized. When analysing BSA-CET molecular complex, it seems to be destabilized by the bonding energy terms, but significantly stabilized by the non-bonding energy components. Nevertheless, being CET a complex molecule with light and heavy chains, future molecular docking studies may better clarify those interactions.

Further in vivo testing is now required to characterize PvD pharmacokinetics and to evaluate its in vivo safety after the nanoencapsulation process. In a preliminary in vivo tolerability assay, a suspension of free PvD in 5% of DMSO (single dose 3 mg / kg) was intradermally injected in a small number of Wistar rats. Plasmatic concentration of PvD increased over the time (up to 6 h) and no animal death was verified till the end of the study (data not shown). In this preliminary study, the intradermal route was chosen mainly due to safety reasons. in vivo efficacy studies using the promising nanoformulation here developed are also warranted.

When analysing ERL and CET conjugated empty and PvD-loaded BSA NPs by DLS, we concluded that ERL conjugated BSA NPs size, zeta potential and PdI values are better aligned with our goals. Accordingly, ERL-CET conjugated BSA NPs showed to have huge particle sizes as well PdI, which leads to a non-monodispersed suspension. On the other side, CET conjugated BSA NPs exhibit sizes that are too small. AFM analysis of those particles suggested a possible interaction between the compounds used that led to some kind of NP undoing process. In addition, CET conjugated BSA NPs showed low toxicity to pancreatic cancer cell lines, with IC_50_ values higher than 30 µM, compared to ERL conjugated BSA NPs, which showed IC_50_ values lower than 20 µM to BxPC3 cell line. Therefore, we choose to proceed our studies only with ERL conjugated BSA NPs. Thus, SEM, TEM, cell cycle and lactate dehydrogenase assays focused on ERL conjugated NPs and non- conjugated NPs. Cell cycle and lactate dehydrogenase assays suggested that the decrease of cell viability caused by NPs forms, as well as by free PvD, may occur by cell cycle arrest in the G1 phase and not by cell death, as membrane integrity remained intact.

## 4. Materials and Methods 

### 4.1. Optimization of BSA-Based NPs Development

Briefly, 50 mg of BSA was dissolved in 2.0 mL of purified water with the pH adjusted to 7–10 with NaOH solution. Subsequently, this solution was added dropwise into 8 mL of absolute ethanol solution under magnetic stirring (100 and 500 rpm). After the desolvation process, the cross-linking agent (glutaraldehyde, glucose, UV light and combination of both glucose and UV light methods) was added to induce particle cross-linking. The cross-linking process was performed under stirring of the suspension over a period of time (30 min and 24 h). First of all, the best stirring rate for NPs formation was defined. Then, after the desolvation process, four cross-linking groups were tested: the first in which 8% glutaraldehyde in water was added to induce particle cross-linking (1.175 μL/mg BSA); the second was placed in the UV chamber under UV light (wavelength 254 nm) for 30 min (the distance between the light source and the sample was 15 cm); on the third, 6 mM glucose was added (1.175 μL/mg BSA); for the forth, the addition of glucose was followed by immediate exposure to UV source in the same conditions mentioned in the second group [43]. After setting these first two steps, cross-linking process was tested under different time periods of stirring. Then, different organic solvents were added to BSA solution for further addition of the active compound in order to verify which one is the best solution for achieving the greatest nanoformulation in terms of size, zeta potential and cross-linking efficacy (CE, %).

### 4.2. NPs Cytotoxicity Assays on Saccharomyces Cerevisiae Model

The use of *S. cerevisiae* as an in vitro model organism has as main advantages the similarity with mammalian cells, the yeast fast growth, and the inexpensiveness and easy cultivation. Yeast is especially useful for toxicity studies involving oxidative stress-related mechanisms, since it can grow both in presence and absence of oxygen [44]. Through this assay, we aimed to study BSA NPs’ potential cytotoxicity, testing the effects on the *S. cerevisiae* growth when contacting with different concentrations of BSA NPs samples. *Saccharomyces cerevisiae* (ATCC^®^ 9763™) was cultivated in yeast extract peptone dextrose medium containing 1% yeast extract, 0.5% peptone and 2% glucose in disposable cuvettes, with a final volume of 2 mL. Non-treated cultures were used as negative control and BSA NPs samples were analysed at different concentrations (3.0 to 5.3 μM). Before adding the BSA NPs to the yeast cultures, an ultrasonic bath (Bandelin SONOREX Super RK510, Berlin, Germany) was used to homogenize the NP suspensions, for 3 min. Then, the assay was carried out as described by Roberto et al. [44]. Briefly, before each absorbance measurements in the spectrophotometer (Thermo Scientific model Evolution 300 BB, Waltham MA, USA) at 525 nm, the cultures were mixed in a vortex. The growth curve in the logarithmic phase for each sample, as well as for the control group was conducted by correlating the logarithm of the cell concentration (number of cells/mL) against the incubation time. Each group was tested in four replicates (n = 4). For each sample and corresponding concentration, the growth inhibition (GI, %) was calculated from the linear slope in the logarithmic phase of *S. cerevisiae* growth curve.

The cell concentrations (cells/mL) were calculated applying Equation (12):

(12) $$n{}^{\circ}\;cells/mL=\;\frac{\left(X*1000mm^{3}\right)}{Y*w^{2}*d}$$

where X = n.º cells counted; Y = n.º of smallest squares counted; d= depth of counting chamber and w= width of 1 square unit [44].

### 4.3. Evaluation of BSA NPs Secondary Structure by far UV Circular Dichroism

Circular dichroism (CD) spectra were recorded on a JASCO J-720 spectropolarimeter (JASCO, Hiroshima, Japan) equipped with a 180–700 nm photomultiplier (EXEL-308). CD spectra were recorded in the far UV range from 260 to 200 nm with quartz Suprasil^®^ CD cuvettes (0.1 cm) at room temperature (ca. 25 °C). Each CD spectrum was the result of six scans recorded in degrees. The following acquisition parameters were used: data pitch, 0.5 nm; bandwidth, 2.0 nm; response, 2 s and scan speed, 50 nm/min.

### 4.4. Haemolytic Activity

The haemolytic activity of PvD in free and encapsulated forms as well as in empty NPs was determined using EDTA-preserved peripheral human blood [36]. Human peripheral blood was obtained from a voluntary donor, then it was EDTA-preserved and used in the same day of experiments. Plasma was removed by centrifugation at 1000× *g* for 10 min and the RBCs suspension was washed three-times in PBS at 1000× *g* for 10 min. The NPs formulations were suspended in PBS buffer (European Pharmacopeia 7.0) and free PvD was dissolved in DMSO and then diluted in PBS. Free PvD concentrations ranging from 0.03 to 51.27 μM and encapsulated PvD concentrations ranging from 0.04 to 80.11 μM were distributed in 96-well plates (100 μL/well). A DMSO control at the higher concentration used for free PvD was also tested. Then, 100 μL of RBCs suspension was added to all samples, microplates were incubated at 37 °C for 1 h and then centrifuged at 800× *g* for 10 min. Absorbance of supernatants was measured at 550 nm with a reference filter at 620 nm. The percentage of the haemolytic activity for each sample was calculated comparing each individual determination to a 100% haemolysis (erythrocytes in distilled water), positive control and negative control (erythrocytes in PBS) according to Equation (13):

(13) (AbsS – AbsN)/(AbsP – AbsN) × 100

where AbsS is the average absorbance of the sample, AbsN is the average absorbance of the negative control and AbsP is the average absorbance of the positive control.

### 4.5. Synthesis of BSA NPs Conjugated with ERL or CET

In this step, we aimed to assess if the EGFR inhibitors and its conjugation with BSA NPs allow or not a targeting deliver of the system to pancreatic tumour cells. Thus, the synthesis of BSA NPs conjugated with ERL or CET was performed by incubating them with a NPs suspension [45,46]. Briefly, EGFR inhibitors (500 µL) were added dropwise into the NPs suspension, followed by a 2 h reaction under stirring (500 rpm). At the end of the reaction, the mixture was centrifuged (2800× *g*/10 min) and resuspended with purified water. The quantification of the EGFR inhibitors conjugation was then determined in the following section.

### 4.6. HPLC Method

A reverse-phase HPLC chromatographic method (stationary phase - LiChrospher RP 18 (5 µm), Lichrocart 250 – 4.6) at a detection wavelength of 254 nm, was used for PvD quantification. Briefly, we used a HPLC system (Hitachi LaCrom Elite, with column oven and a UV-Vis diode array detector, San Jose CA, USA) with a mobile phase containing methanol and trichloroacetic acid 0.1% (80:20, v/v) at a flow rate of 1.0 mL/min. The injection volume was 20 μL, the run-time was 15 min and the column conditions were maintained at 30 °C. Measurements were carried out in duplicate and PvD encapsulation efficiency was calculated according to Equation (14):

EE (%) = (Amount of encapsulated drug/Initial drug amount) × 100(14)

For ERL quantification, a reverse-phase HPLC chromatographic method on a NovaPak^®^ C18 column (3.9 × 150 mm) from Waters (Milford, MA, USA) was used, at a detection wavelength of 346 nm, as previously described [47]. Briefly, the same HPLC system was used but with an isocratic mobile phase consisting of acetonitrile and acidified water pH 2.6 (40:60, v/v) and a flow rate of 1.0 mL/min. Column conditions were maintained at 22 °C, with an injection volume of 20 μL and a retention time of 4.5 min. Measurements were carried out in duplicate.

### 4.7. LC-MS/MS Analysis for CET Quantification

LC-MS/MS analyses were performed as previously described by François et al. [48]. Briefly, a XEVO TQ-S triple quadrupole mass spectrometer (Waters) was used, controlled by Masslynx software (version 4.1) and an Acquity I-Class LC system (Waters) was linked to the instrument. Chromatography was performed using a gradient combining 0.1% formic acid in water with 0.1% formic acid in acetonitrile. Peptides separation was achieved on an Acquity UPLC BEH Shield C18 column, 2.1 mm × 50 mm, 1.7 μm, (Waters), using a gradient from 5 to 33% over 10 min, at a flow rate of 500 μL/min and the temperature was maintained at 50 °C.

### 4.8. Static Lattice Atomistic Simulations

Molecular simulations were performed using commercial software: HyperChemLite^TM^ 8.0.8 Molecular Modeling System (Hypercube Inc., Gainesville, FL, USA) and ChemBio3D Ultra 11.0 (CambridgeSoft Corporation, Cambridge, UK). The structures of glucose and ERL were with natural angles as a 3D model while the structures of the peptide chains BSA (BSA; sequence: LQARILAVERYLKDQQL) and CET (CET; PDBID:4GW5; Chain A) were generated using the built-in sequence editor module of HyperChem. A progressive-convergence-strategy was applied for energy minimization with MM+ force field. The confirmers having lowest energy were employed for geometrical optimization and the molecular complexes were assembled by parallel disposition to generate the final models: BSA-glucose, BSA-ERL, and BSA-CET corresponding to BSA formulations with glucose cross-linking and ERL/CET functionalization, respectively. Full geometry optimization was carried out in vacuum employing the Polak–Ribiere conjugate gradient algorithm until an RMS gradient of 0.001kcal/mol was reached. For molecular mechanics calculations in vacuum, the force fields were utilized with a distance-dependent dielectric constant scaled by a factor of 1. The 1–4 scale factors were electrostatic = 0.5 and van der Waals = 0.5 [49].

### 4.9. Physical and Morphological Characterization of the EGFR Inhibitors Conjugated BSA NPs (Size, Surface and Morphology): Dynamic Light Scattering (DLS) and Atomic Force Microscopy (AFM), Scanning Electron Microscopy (SEM) and Transmission Electron Microscopy (TEM)

Freshly prepared nanoformulations were studied in terms of their structure, surface morphology, shape and size by DLS, AFM, SEM and TEM. The analysed samples were: empty BSA-NPs, PvD-loaded BSA NPs, ERL conjugated empty BSA NPs, ERL conjugated PvD-loaded BSA NPs, CET conjugated empty BSA NPs, CET conjugated PvD-loaded BSA NPs, ERL-CET conjugated empty BSA NPs ERL-CET conjugated PvD-loaded BSA NPs.

DLS (Coulter nano-sizer Delta Nano™, Brea, CA, USA) was used to perform a physical characterization of the NPs, evaluating mean particle size, polydispersity index (PdI) and zeta potential of the NPs’ diluted suspension (n = 3). For DLS analysis, samples were diluted with purified water (1:10) and the measurements were carried out at room temperature (25 °C).

An atomic force microscope, Multimode 8 coupled to Nanoscope V Controller (Bruker, Coventry, UK) was used to acquire AFM images by using peak force tapping and ScanAssist mode. An aliquot of each sample (~30 μL) was mounted on a freshly cleaved mica sheet and left to dry before being analysed, in order to offer a clean and flat surface for AFM analysis. The images were obtained in ambient conditions, at a sweep rate close to 1 Hz, using Scanasyst-air 0.4 N/m tips, from Bruker.

For SEM, all samples were fixed with 2.5% glutaraldehyde in 0.1 M sodium phosphate buffer (European Pharmacopeia 7.0), at pH 7.2 during 1 h. After centrifugation, the pellets were washed three times in the fixative buffer and aliquots (10 μL) of the sample suspensions were scattered over a round glass coverslip previously coated with poly-L-lysine. The samples were then exposed to osmium tetroxide vapours in a fixative chamber during 30 min, dehydrated in a graded series of ethanol and dried with hexamethyldisilazane. After coated with a thin layer of gold, the samples were observed on a JEOL 5200LV scanning electron microscope (JEOL Ltd., Tokyo, Japan) at an accelerating voltage of 20 kV, and images were recorded digitally.

For TEM, the samples were fixed with glutaraldehyde as for SEM and aliquots (10 μL) of the samples’ suspensions were dispersed on carbon-coated copper grids. Afterwards, the material was negative staining with 1% uranyl acetate and left to dry at room temperature. Observations were carried out on a JEOL 1200EX transmission electron microscope (JEOL Ltd.) at an accelerating voltage of 80 kV. Images were recorded digitally.

### 4.10. Cell Viability Studies of PvD-Loaded BSA NPs

In order to evaluate PvD selectivity and antiproliferative effects against human pancreatic tumour cells in its free form, as well as when encapsulated in BSA NPs, two different cell lines were tested, BxPC3 and PANC-1 (human pancreatic adenocarcinoma cell lines). Both BxPC3 and PANC-1 cell lines were obtained from the ATCC (Manassas, VA, USA).

The cell lines tested are typically adherent cell cultures. Evaluations were made in different experimental conditions regarding the different types of cells. Thus, BxPC3 cells were maintained in in RPMI 1640 medium with glutamax, supplemented with 10% (v/v) inactivated fetal bovine serum (iFBS), 50 U/mL penicillin and 50 μg/mL streptomycin. PANC-1 cells were maintained in Dulbecco’s Modified Eagle’s medium (DMEM) with high-glucose (4500 mg/L), supplemented with 10% iFBS, 50 U/mL penicillin and 50 μg/mL streptomycin. Both cell lines were maintained in an incubator at 37 °C in a humidified atmosphere of 5% CO_2_.

The effect of PvD on cell proliferation was evaluated by MTT assay [50]. Briefly, cells were seeded in 96-well plates (1 × 10^4^ cell per well for BxPC3 and for PANC-1) under normal conditions (5% CO_2_ humidified atmosphere at 37 °C) and allowed to adhere overnight. The cells were then incubated for 48 h with free PvD, ERL conjugated empty BSA NPs, ERL conjugated PvD-loaded BSA NPs, CET conjugated empty BSA NPs, CET conjugated PvD-loaded BSA NPs, ERL-CET conjugated empty BSA NPs, ERL-CET conjugated PvD-loaded BSA NPs, PvD-loaded BSA NPs and Empty BSA NPs at different concentrations: between 5 and 30.0 µM for BxPC3 and to PANC-1 between 10.0 and 40.0 µM for all PvD-loaded nanoformulations and free PvD and between 20 and 30.0 µM for BxPC3 and to PANC-1 between 30.0 and 40.0 µM for all empty nanoformulations. The cells medium was removed after the incubation period and PBS was used to wash the wells. Then, MTT solution (50 µL of a 10%) was added to the cells and the plates were incubated for a 4 h period. After the incubation time, to solubilize the formazan crystals formed during the incubation period, DMSO was added to each well (100 µL).

Using a microplate reader, it was measured the absorbance of all samples at 570 nm and the IC_50_ was determined. The cytotoxic effect was evaluated by determining the percentage of viable/death cells for each formulation studied concentration. Cell proliferation analysis was carried out in GraphPad Prism^®^5 (GraphPad Software, San Diego, CA, USA). Values were plotted and fit to a standard inhibition log dose-response curve to generate the IC_50_ values. A total of three independent experiments, with six replicates per condition, were carried out.

### 4.11. Cell Cycle Assays

BxPC3 human pancreatic carcinoma cells were used as a tumour cell model to evaluate the effects of PvD-loaded NPs and PvD in its free form on cell cycle progression. Cell cycle progression was evaluated using a standard staining procedure with propidium iodide (PI) (Fluka, Sigma-Aldrich, Darmstadt, Germany) followed by flow cytometry. Briefly, BxPC3 cells were incubated with free PvD, PvD-loaded BSA NPs and ERL conjugated PvD-loaded BSA NPs at a PvD concentration of 10 µM for 24 h. Next, cells were detached with Tryple and collected by centrifugation at 800× *g* for 5 min, at 4 °C. Cell pellets were resuspended in ice-cold PBS and fixed under gentle vortexing by dropwise addition of an equal volume of ice-cold 80% ethanol (−20 °C), followed by 30 min on ice. Subsequently, samples were stored at 4 °C for at least 18 h until data acquisition. For cell cycle analysis, cells were centrifuged at 850× g for 5 min, at 4 °C, and pellets resuspended in RNase A (50 μg/mL, in PBS) and PI (25 μg/mL) and further incubated for 30 min, at 37 °C. Sample acquisition and data analysis were performed using the Guava easyCyte^TM^ Flow Cytometer (Merck Millipore, Darmstadt, Germany) and Guava analysis software, with the acquisition of at least 10,000 events per sample.

### 4.12. Lactate Dehydrogenase (LDH) Assay

The lactate dehydrogenase assay measures membrane integrity as a function of the amount of cytoplasmic LDH released into the medium. The assay is based on the reduction of NAD by LDH. Reduced NADH is then utilized in the conversion of a tetrazolium dye to a coloured product that is measured spectrophotometrically. The 24 h incubation medium collected in the cell cycle assay was used to perform this assay for the same samples used in the previous assay: free PvD, ERL PvD-loaded BSA NPs and ERL conjugated PvD-loaded BSA NPs at a PvD concentration of 10 µM. A 50 μL aliquot of each incubation medium was collected and transferred to a 96-well plate and 50 μL of Lactate Dehydrogenase Assay Mixture was added and gently mixed. The plate was covered with an opaque material to protect from light and incubated at room temperature for 10 min. Samples absorbance was measured at a wavelength of 490 nm in a MR 4000 plate reader (Model 680 Microplate reader, Bio-Rad Laboratories, Hercules, CA, USA).

### 4.13. Statistical Analysis

The significance of differences between samples was assessed using one-way analysis of variance (ANOVA) for mean comparisons (Tukey’s test). A 0.05 significance level was adopted for every test.

## 5. Conclusions

BSA NPs were efficiently produced and PvD was successfully encapsulated. The BSA secondary structure was shown to be maintained after NP formation and after submitting BSA NPs to ultra-sounds and heat, which can be seen as verification of the formation of stable nanocarriers. Regarding haemolytic activity, PvD-loaded BSA NPs have shown to be safe to human RBCs, which allows this formulation to be administered in future injectable use. Static lattice atomistic simulations have proven that these nanocarriers are a stable formulation, suggesting that glucose was an excellent choice as a cross-linking agent, with lower toxicity than glutaraldehyde. Concerning physical and morphological characterization of the NPs there are considerable differences between all the formulations developed, being ERL conjugated BSA NPs and non-conjugated BSA NPs (empty and PvD-loaded) the formulations that have shown better results according to our goals: size ranging between 100–400 nm, with monodisperse size distribution, well-defined morphology, high cross-linking efficacy and negative zeta potential. Cell culture and cytotoxicity assays indicated that NPs can damage pancreatic tumour cells. However, the results of CET conjugated NPs, when comparing to ERL conjugated NPs, showed that ERL conjugated NPs were more efficient at reducing cell viability. Thus, ERL conjugated NPs were chosen to proceed to SEM and TEM analysis, as well as to cell cycle and lactate dehydrogenase assays. This formulation showed well-defined morphology by SEM and TEM analysis. Since there was no significant damage in cell membrane integrity, our results suggest that the decrease on cell viability by these particles may occur, at least in part, by a cell cycle arrest-dependent mechanism rather than by inducing cell death. 

Further studies must be undertaken, including mechanistic studies of the optimized formulation, by performing in vivo assays to test ERLconjugated PvD-loaded BSA NPs targeting efficacy as well as safety.

## Figures and Tables

**Table 1:** BSA nanoparticle (NP) characterization in terms of size, zeta potential, polydispersity index (PdI) and cross-linking efficacy (CE (%))—influence of experimental conditions.

		Mean Size (nm) (± SD)	Zeta Potential	PdI	CE (%)
**Cross-linking agent**	Glutaraldehyde	178 (± 43)	−1	<0.220	54
	**Glucose**	**225 (± 60)**	**−14**	**<0.250**	**97**
	Glucose + UV	237 (± 70)	−4	<0.120	44
	UV	600 (± 116)	−1	<0.180	95
**Stirring rate** **(rpm)**	100	4396 (± 398)	0	<0.710	98
	**500**	**225 (± 25)**	**−14**	**<0.250**	**97**
**Cross-linking time**	24 h	129 (± 34	11	<0.100	56
	**30 min**	**225 (± 25)**	**−14**	**<0.250**	**97**
**Type of organic solvent**	DMSO	153 (± 25)	0	<0.010	69
	Ethanol	145 (±42)	−2	<0.330	50
	**Acetone**	**244 (± 9)**	**−8**	**<0.260**	**98**
	Hexane	1346 (± 144)	−1	<0.180	88
**Aqueous: Organic ratio**	1:1	236 (± 62)	−1	<0.230	78
	2:1	898 (± 33)	0	<0.210	77
	**3:1**	**244 (± 9)**	**−8**	**<0.260**	**98**

**Table 2:** Particle size, polydispersity index (PdI) and zeta potential of all EGFR inhibitors conjugated BSA nanoformulations by DLS analysis.

BSA Nanoformulations	Mean Diameter (nm) ± SD	Mean Zeta Potential (mV) ± SD	PdI
ERL-CET conjugated PvD-loaded BSA NPs	1466 (±155)	−48 (± 6)	<0.560
ERL-CET conjugated empty BSA NPs	502 (± 36)	−32 (± 6)	<1
ERL conjugated PvD-loaded BSA NPs	349 (± 59)	−39 (± 10)	<0.450
ERL conjugated empty BSA NPs	336 (± 91)	−36 (± 5)	<0.090
CET conjugated PvD-loaded BSA NPs	43 (± 4)	−43 (± 4)	<1
CET conjugated empty BSA NPs	42 (± 5)	−32 (± 6)	<1
PvD-loaded BSA NPs	280 (± 86)	−42 (± 4)	<0.370
Empty BSA NPs	393 (± 131)	−37 (± 6)	<0.120

**Table 3:** Antiproliferative effect (IC_50_) of PvD in free form or nanoformulated in two human pancreatic adenocarcinoma cell lines (BXPC3 and PANC-1).

Formulation Tested	BxPC3	PANC-1
Free PvD	10.6 ± 3.6	21.7
Empty BSA NPs	>30	>40
PvD-loaded BSA NPs	>30	>40
ERL conjugated empty BSA NPs	<20	>40
ERL conjugated PvD-loaded BSA NPs	21.5 ± 2.2	16.8
CET conjugated empty BSA NPs	>30	>40
CET conjugated PvD-loaded BSA NPs	>30	>40
ERL-CET conjugated empty BSA NPs	<20	>40
ERL-CET conjugated PvD-loaded BSA NPs	6.9 ± 1.1	> 40

Incubation period was 48 h. IC_50_ values are expressed in µM (n = 6).
